# pH-responsive intermediary layer cross-linked micelles from zwitterionic triblock copolymers and investigation of their drug-release behaviors

**DOI:** 10.55730/1300-0527.3597

**Published:** 2023-09-30

**Authors:** Agung Ari WIBOWO, Vural BÜTÜN

**Affiliations:** 1 Department of Polymer Science and Technology, Institute of Science, Eskişehir Osmangazi University, Meşelik Campus, Eskişehir, Turkiye; 2 Department of Chemistry, Faculty of Science, Eskişehir Osmangazi University, Eskişehir, Turkiye

**Keywords:** Atom transfer radical polymerization, pH-responsive polymer, zwitterionic polymer, micelles, cross-linked micelles, drug release

## Abstract

ABC-type triblock copolymers, namely poly[(ethylene glycol)methyl ether]-*block*-poly(*tert*-butyl methacrylate)-*block*-poly[2*-N-*(diisopropylamino)ethyl methacrylate] (MPEG-*b*-PBuMA-*b*-PDPA), were first synthesized and then the middle blocks were successfully converted into poly(methacrylic acid) to obtain MPEG-*b*-PMAA-*b*-PDPA zwitterionic triblock copolymers. These block copolymers were soluble in water and formed micellar aggregates with complex cores via hydrogen bonding interactions between MPEG and PMAA blocks below pH 4.0. When the pH was between 5.0 and 7.0, due to charge compensation between partially protonated PDPA and partially ionized PMAA blocks, micelles with polyion complex cores were observed. If the solution pH was above 8.0, deprotonation of tertiary amine groups provided a hydrophobic character to the PDPA block, which resulted in the formation of PDPA-core micelles while MPEG/anionic PMAA hybrid blocks formed hydrated coronas. Intermediary layer cross-linked (ILCL) micelles from PDPA-core micelles were also prepared by cross-linking the inner PMAA shell. The hydrophobic drug dipyridamole (DIP) was used to investigate the release profile of ILCL micelles. DIP can be loaded to the PDPA cores of the micelles in basic aqueous media. An increase in the degree of cross-linking causes slower release for the model drug. It was concluded that the more complex matrix formation in the intermediary layer of the micelles via cross-linking retards the drug release from the core.

## 1. Introduction

Developments in targeted drug delivery to specific organs and the increasing of its efficiency have attracted great interest. To develop new vehicles to be used in drug delivery that improve the therapeutics index and bioavailability, the combination of multidisciplinary sciences is needed [1]. The purposes of drug delivery systems being incorporated with existing medicines are to improve their efficacy, safety, and patient compliance [2]. Applications of nanomaterials in the field of medicine including tissue-engineered scaffolds and devices, specific site-targeting delivery systems, and cancer therapy reflect the significant developments in this area [2–4]. With their wide application fields, polymers are becoming important in drug delivery technology, especially in terms of their usage as drug carrier vehicles. To improve drug stability in plasma and to modify the release, polymers can be used as film coatings [4].

Nanoparticles such as polymeric micelles have the potential to become drug vehicles and can likely enhance the performance. The classic problem in using micelles as drug delivery vehicles is micelle degradation upon changes in environmental conditions within the plasma protein, which can be caused by pH, temperature, or dilution under the critical micelle concentration. Thus, compact and stabilized micelle structures are required for related applications. The development of polymer synthesis, cross-linking, and functionalization of polymer micelles has accordingly attracted great interest [5–7]. In terms of stabilizing polymeric micelles with cross-linking, it is possible to obtain various types of cross-linked micelles (CLMs) with different methods including core cross-linking, shell cross-linking [6], and intermediary layer cross-linking (ILCL) chemistry [8,9].

Recently, various research groups have reported a number of studies on the potential usage of stimuli-responsive CLMs based on diblock and triblock copolymers as drug delivery vehicles [10–20]. In the case of diblock copolymer usage [10–15], CLM syntheses using amphiphilic diblock copolymers for drug transport and release are concentrated on core cross-linked structures, since the diluteness of such polymeric micelles in corona cross-linking is an important limitation in terms of application. Triblock copolymers are highly suitable structures for CLM synthesis without inter-micelle cross-linking at high micelle concentrations. In this case, the hydrophobic core or hydrophilic inner layer can be cross-linked at high polymeric micelle concentrations [16].

As a thermo-responsive triblock copolymer, He et al. [18] reported a series of poly(ethylene oxide)monomethyl ether-*b*-poly(acrylic acid)-*b*-poly(N-isopropylacrylamide) (PEO-*b*-PAA-*b*-PNIPAM) triblock copolymers. After the loading of DOX into the PNIPAM core of the micelles, the cross-linking of the inner PAA blocks was carried out using 1-(3-dimethylaminopropyl)-3-ethyl carbodiimide hydrochloride and 2,2′(ethylenedioxy)bis(ethylamine). A release study was performed at pH 7.4 and temperatures of 37 °C and 25 °C. They concluded that DOX-loaded CLMs exhibited thermo-responsive drug release behavior, and cross-linking had an important effect on the drug release behavior of the system. Another temperature- and pH-sensitive triblock copolymer, namely poly(ethylene oxide)monomethyl ether-*b*-poly(N-(3-aminopropyl)methacrylamide)-*b*-poly(N-isopropylacrylamide) (PEO-*b*-PAPMA-*b*-PNIPAM), and its ILCL micelles were reported by Xu et al. [19]. Cross-linking of the inner PAPMA shell was carried out with terephthaldicarboxaldehyde in a basic solution to get CLMs with imine linkages. Such cross-linking is defined as cleavable cross-linking, which can be reversibly broken with stimuli [16–18]. After loading a model drug into CLMs, the drug is released by breaking the imine bond reversibly in acidic medium. These researchers concluded that such cross-linked micelles had promise as therapeutic nanocarriers in biomedicine. In another study, a PDEA-*b*-PDMA-*b*-PNIPAM block copolymer that was both pH- and thermo-responsive was reported by Jiang et al. [20]. This block copolymer can form PNIPAM-core micelles in acidic solution and PDEA-core micelles at basic pHs at room temperature. The inner PDMA blocks were then cross-linked to get dual-responsive ILCL micelles. The dipyridamole (DIP) drug release behavior of these PDEA-core ILCL micelles indicated that drug release was greater at both acidic pH levels and body temperature.

Other pH-responsive ILCL micelles for drug delivery were reported by Aydın [21]. After the synthesis of end-functional PBA, PEO, and PMMA homopolymers, an amphiphilic triblock copolymer was successfully synthesized by conjugating them with sequential “click” reactions. The middle block of the precursor PEO-PBA-PMMA was first converted to PAA, followed by modification with *Boc*-protected hydrazine and finally acid hydrolysis to obtain acyl hydrazide functionality, which can be reacted with a glutaraldehyde cross-linker to form an acid-sensitive hydrazone linkage. The final PEG*-b-*PAH*-b-*PMMA block copolymer was used to prepare drug-encapsulated CLMs in aqueous media. The pH-sensitive hydrazone linkage in the middle layer allowed the control of the release rate of the encapsulated model drug. Another cross-linking method to cross-link the PAA blocks of block copolymers is physical complex formation with a metal cation. Cross-linking can be accomplished simply by adding a metal cation to form complex PAA-M^n+^ cores [22]. Similarly, there are various reports based on cross-linked ionic micelles including polyzwitterionics [23] and polybetaines [24]. However, as drug delivery and release systems, cross-linked micelles still have limitations in terms of effective drug transport, release control, and biocompatibility.

Herein, we report the synthesis of MPEG-*b*-PBuMA-*b*-PDPA triblock copolymers followed by acid hydrolysis of the middle PBuMA blocks to zwitterionic MPEG-*b*-PMAA-*b*-PDPA triblock copolymers. These triblock copolymers have a pH-responsive nature and can self-assemble to form different types of micelles. MPEG is a well-known hydrophilic polymer in drug carrier vehicles. PMAA is an ionizable hydrophilic polymer and has a pK_a_ value of 5.5. PDPA is a weak polybase with a pK_a_ of 6.4 [25]. As mentioned above, to be suitable drug delivery vehicles, micelles should be stabilized well to avoid destabilization by enzymes or changes in environmental conditions. In our system, PMAA blocks provide an advantage for preparing cross-linked MPEG-*b*-PMAA-*b*-DPA micelles on a relatively short time scale by using a 1‚2-bis-(2-iodoethoxy)ethane (BIEE) cross*-*linker. The cross-linking reaction of PMAA with BIEE in aqueous medium was first reported by us [26] and determined to be faster than cross-linking over PDMA residues. This gives a great advantage in terms of the encapsulation of the drugs by shortening the loading and encapsulation periods. Our aim was to shorten the cross-linking period of drug-loaded micelles and to tune the release profile of CLMs by varying degrees of cross-linking. In pursuing this aim, we paid attention to the drug release profile of both precursor micelles and CLMs within a specific period.

## 2. Experimental section

### 2.1. Materials and chemicals

*Tert*-butylmethacrylate (*t*BuMA) was purchased from Tokyo Chemical Industry and 2-*N*-(diisopropylamino)ethyl methacrylate (DPA) was purchased from Aldrich. Hydroquinone inhibitors from the monomers were removed by passing them through a basic alumina column and the monomers were stored at −20 °C. A macroinitiator (MPEG-Br) was synthesized using poly(ethylene glycol)methyl ether (MPEG, M_n_ = 2000 g/mol, Fluka) and α-bromoisobutyryl bromide (BIBB, Aldrich) in the presence of triethylamine (TEA, Merck). 1,2-Bis(2-iodoethoxy)ethane (BIEE) cross-linker was purchased from Aldrich. Copper(I) bromide (CuBr) was purchased from ChemPUR and 2,2′-bipyridine (Bpy) was obtained from Alfa Aesar.

### 2.2. Synthesis

#### 2.2.1. Synthesis of MPEG-Br macroinitiator

A known amount of methyl-ether polyethylene glycol (MPEG, M_w_ = 2000 g/mol, 20 g) was introduced to a round-bottom flask. Toluene (100 mL) was then added and the mixture was stirred for 20 min to ensure the complete dissolution of MPEG. TEA (2.6 mL) was then introduced to the flask prior to the addition of BIBB (2.3 mL). The reaction was continued for the next 72 h and then the formed salt was filtered. The remaining solvent was evaporated using a rotary evaporator until a viscous solution was obtained. The MPEG-Br was precipitated in diethyl ether (500 mL) and washed two times before being dried under vacuum for 24 h.

#### 2.2.2. Synthesis of MPEG-*b*-PMAA-*b*-PDPA triblock copolymer

The MPEG_45_-*b*-PMAA_47_-*b*-PDPA_47_ triblock copolymer was synthesized via atom transfer radical polymerization (ATRP). All steps were carried out under inert nitrogen gas. First, the well-defined MPEG-Br (2.0 g) macroinitiator, CuBr (0.134 g), and Bpy (0.29 g) were introduced to a 50-mL round-bottom flask and degassed by purging nitrogen. In the other containers, 15 mL of anisole and 7.5 mL of *t*BuMA were also degassed separately. After 15 min, degassed anisole was transferred to the flask via a cannula. The heating of the solution in a certain short period of time was needed because MPEG-Br had hardly dissolved. The solution turned dark brown and was stirred for 15 min to ensure the complete dissolution of MPEG-Br. The degassed *t*BuMA was then transferred to the reaction flask using a cannula. After 24 h, a small amount of aliquot (0.3 mL) was taken from the flask to be analyzed by gel permeation chromatography (GPC) as a diblock sample. The degassed mixture of anisole (10 mL) and DPA (10.8 mL) was then transferred to the flask to get the third block and the reaction was continued for the next 24 h. The polymerization reaction was terminated by treating it with atmosphere and it was diluted in acetone. The catalyst was removed using a basic alumina column and the resulting solution was concentrated via evaporation with a rotary evaporator, followed by precipitation twice in THF to remove the unreacted macroinitiator and trace catalyst contaminants and before freeze-drying for 24 h.

The *t*-butyl groups in the middle blocks of the block copolymers were removed using concentrated HCl in THF at room temperature for 72 h. After evaporating the solvent until the solution became viscous, the residue was precipitated in diethyl ether. The final product was freeze-dried for 24 h. The yield was quantitative (98%). The MPEG_45_-*b*-PMAA_20_-*b*-PDPA_18_ triblock copolymer was synthesized by changing the amounts of MPEG_45_-Br (4.0 g), CuBr (0.26 g), and Bpy (0.58 g) with the same procedure described here.

#### 2.2.3. Preparation of micelles and ILCL micelles

The MPEG*-b-*PMAA*-b-*PDPA triblock copolymer was first dissolved in distilled water (1.0 w%). The adjustment of the solution pH to 10.0 was carried out using 1.0 M NaOH. Analysis of proton nuclear magnetic resonance (NMR) spectroscopy and dynamic light-scattering (DLS) studies indicated PDPA-core micelle formations. Inner shell cross-linking at various targets based on PMAA blocks was achieved at pH 10.0 and 25 °C in 2 h using BIEE as the cross-linking agent.

#### 2.2.4. Preparation of drug-loaded ILCL micelles

DIP (15 mg) was dissolved in distilled water (5 mL) at pH 3.0 and stirred for 10 min to ensure full dissolution of the DIP. In another container, 0.4 g of the triblock copolymer was dissolved in 35 mL of water. The drug solution was then added to the polymer solution until the polymer concentration reached 1.0 w%. After adjusting the solution pH to 10.0, it was stirred for 24 h at 1250 rpm and room temperature. For equilibrium, the drug-loaded micellar solution was left steady for 20 min at 25 **°**C before filtering it with a 0.45-μm Millipore filter to remove any nonencapsulated DIP. The maximum capacity of the drug encapsulation was determined using UV-Vis absorbance analysis of the filtrate at 283.52 nm based on a standard curve for DIP. The solution was then divided into four equal parts (10 mL each), and a cross-linking reaction was carried out using the BIEE cross-linker by stirring for 2 h. The targeted degrees of cross-linking were 0%, 20%, 40%, and 60% based on PMAA residues. At the same time, in vitro drug release studies were carried out by taking 2 mL of each drug-loaded ILCL micellar solution into the dialysis membrane (MWCO: 14000 g/mol) before immersion in 200 mL of phosphate buffer solution (pH 7.4) at 37 **°**C. Aliquots (2 mL) were withdrawn from the solution periodically and then diluted with water and adjusted back to pH 3.0. To keep the solution volume constant, 2 mL of phosphate buffer solution was added after each sampling. The released amount of DIP from the micelles was measured using UV absorbance at 283.52 nm.

### 2.3. Copolymer characterizations

#### 2.3.1. Gel permeation chromatography (GPC)

Molecular weights (*M_n_*) and molecular weight distributions (*M_w_/M_n_*) of all nonionic precursor (co)polymers were determined by GPC (Agilent 1200 series GPC). The columns were mixed ‘D’ and ‘E.’ PMMA standards with *M_n_* ranging from 650 to 22000 g/mol were used for calibration. The GPC eluent was HPLC-grade THF (Aldrich) stabilized with BHT (2,6-di-t-butyl-4-methyl phenol, Fluka) at a flow rate of 1.0 mL min^−1^.

#### 2.3.2. Nuclear magnetic resonance (NMR) spectroscopy

A Bruker AC-P 300 MHz NMR instrument was used to determine the compositions of the triblock copolymers. The NMR solvent was either DMSO or CDCl_3_. The –OCH_3_ signal at δ 3.3–3.4 due to the macroinitiator fragment was used to calculate the actual degree of polymerization (DP) of both the PBuMA and PDPA blocks. After hydrolysis, the triblock copolymer compositions were determined using DMSO-d_6_ as a solvent. ^1^H NMR spectroscopy studies were also carried out to characterize the micellization behavior of the block copolymers in D_2_O. The solution pH was varied with DCl and NaOD additions.

#### 2.3.3. Dynamic light-scattering Studies (DLS)

Hydrodynamic radii and the polydispersity indexes (PDIs) of the micelles were determined via DLS (ALV/CGS-3 Compact Goniometer System, Malvern). Triblock copolymer solutions of 1% (w/v) were used in all measurements at 20 °C. The scattering angle was fixed to 90° and second-order cumulant analysis was chosen for the data fitting.

#### 2.3.4. Zeta potential measurements

Electrophoretic mobility measurements were obtained with a Zetasizer Nano ZS instrument (Malvern Instruments). The results were confirmed by making three repetitive measurements. The zeta potential values of both uncross-linked micelles and ILCL micelles were determined as a function of solution pH and the targeted degree of cross-linking. In the zeta potential measurements, as in DLS studies, the micelle and ILCL micelle concentrations were adjusted to 1.0% (w/v).

#### 2.3.5. Transmission electron microscopy (TEM) and UV-Vis spectrophotometry

In order to examine the morphology of the ILCL micelles, TEM was used. Dilute (<0.1%) suspensions of the cross-linked micelles were dried onto a 200-mesh formvar/carbon-supported copper grid (TED PELLA-01801) and examined using a Jeol JEM-1220 instrument without any staining. The rates of drug release from the micelles and encapsulated drugs were determined using a PerkinElmer UV/VIS Lambda 35 spectrophotometer. Absorption was measured at 283.52 nm.

## 3. Results and discussion

### 3.1. Synthesis and characterization of MPEG*-b-*PMAA*-b-*PDPA triblock copolymers

**Scheme 1:**
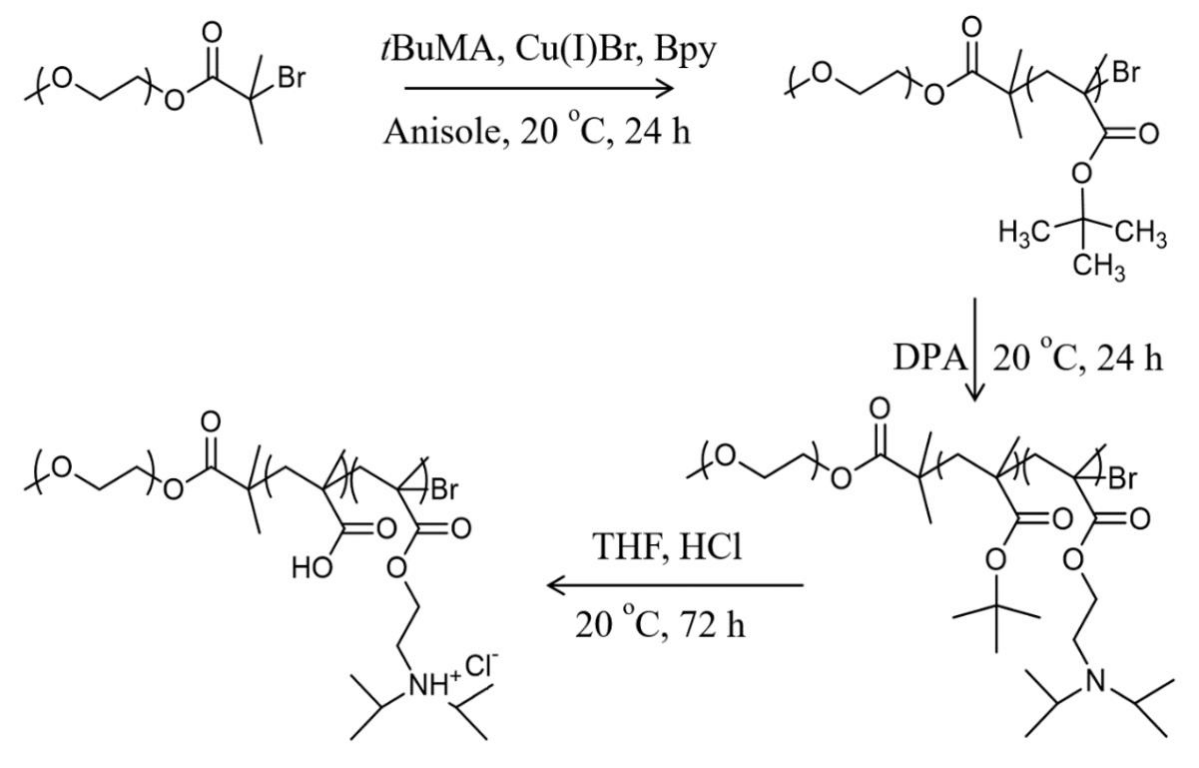
Pathway for the synthesis of MPEG-*b*-PMAA-*b*-PDPA triblock copolymer.

To obtain the pH-responsive MPEG-*b*-PMAA-*b*-PDPA triblock copolymers, MPEG-*b*-PBuMA-*b*-PDPA copolymers as precursors were synthesized by ATRP using MPEG-Br as a macroinitiator followed by acid hydrolysis in THF (see Scheme 1). The MPEG-Br was first prepared through the esterification reaction of the MPEG with 2-bromoisobutyryl bromide (BIBB).

**Figure 1:**
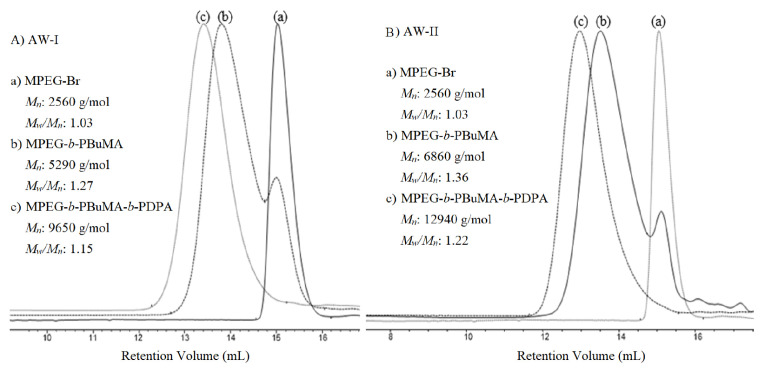
GPC chromatogram from each step in the synthesis of (A) MPEG_45_-*b*-PBuMA_20_-*b*-PDPA_18_ and (B) MPEG_45_-*b*-PBuMA_47_-*b*-PDPA_47_ triblock copolymers.

The esterification of MPEG with BIBB was conducted in toluene in the presence of TEA. An excess of 10% BIBB was used to achieve the complete transformation of the end group. The *M*_n_ and *M_w_/M_n_* values determined by GPC are summarized in the Table. The GPC trace of each step in the synthesis of MPEG-*b*-PBuMA-*b*-PDPA is shown in Figure 1.

**Figure 2:**
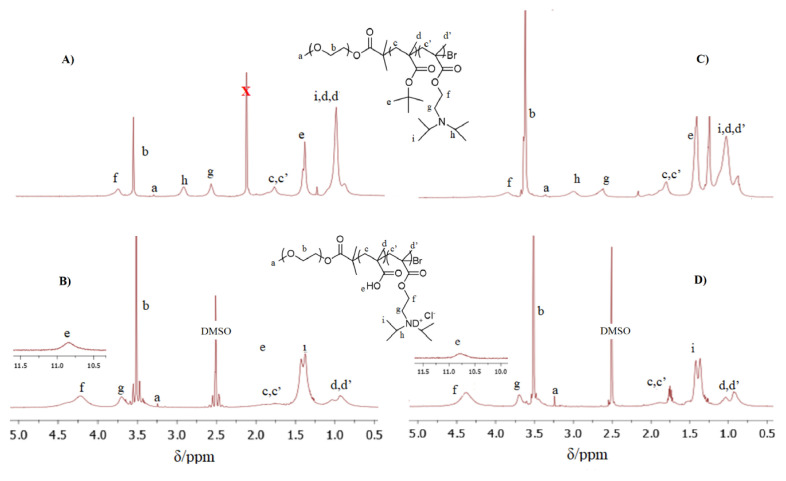
^1^H NMR spectra of triblock copolymers: MPEG_45_-*b*-PBuMA_20_-*b*-PDPA_18_ in CDCl_3_ (A), MPEG_45_-*b*-PMAA_20_-*b*-PDPA_18_ in DMSO (B), MPEG_45_-*b*-PbuMA_47_-*b*-PDPA_47_ in CDCl_3_ (C), and MPEG_45_-*b*-PMAA_47_-*b*-PDPA_47_ in DMSO (D).

The comonomer compositions of the triblock copolymers were calculated using ^1^H NMR spectra taken in CDCl_3_. End-group analysis was carried out by comparing the CH_3_–protons of MPEG at δ 3.3 ppm with both peak integrals of 9 protons of the *tert*-butyl group in the PBuMA residue at δ 1.4 ppm and the peak integral of 12 protons of diisopropylamino groups of the DPA residue at δ 1.1 ppm (Figure 2a and 2b). The degrees of polymerizations of each block of the MPEG-*b*-PBuMA-*b*-PDPA block copolymers were calculated from ^1^H NMR spectra to be 45/20/18 and 45/47/47, respectively.

After acid hydrolysis in THF with concentrated HCl for 72 h, the products of MPEG-*b*-PMAA-*b*-PDPA were analyzed by ^1^H NMR in DMSO-d_6_. The ^1^H NMR spectrum of each step of the MPEG-*b*-PMAA-*b*-PDPA synthesis is shown in Figure 2. Based on comparison with the former spectrum, a new peak at δ 10.75 ppm appeared. This peak indicated the protons of the carboxyl groups (–COOH) in PMAA blocks. The disappearance of the signal at δ 1.4 ppm assigned to the –C(CH_3_)_3_ from PBuMA was also an indication of the removal of the *tert*-butyl groups. Instead of the proton from the *tert*-butyl group, the peak at δ 1.4 ppm came from 12 protons of the diisopropylamino groups of the DPA residue (Figures 2c and 2d). This result indicated that hydrolysis was successfully carried out to transform the MPEG-*b*-PBuMA-*b*-PDPA to the MPEG-*b*-PMAA-*b*-PDPA structure.

### 3.2. pH-induced micellization of the MPEG-*b*-PMAA-*b*-PDPA triblock copolymer

As is generally known, MPEG is a water-soluble and hydrophilic polymer. PMAA is a weak acidic hydrophilic polymer with a pK_a_ value of about 5.5; it is less soluble in acidic solutions but soluble as an anionic polyelectrolyte in basic solutions [27]. On the other hand, PDPA is a weak polybase with a pK_a_ of 6.4 [25,28]. In acidic solutions (pH < pK_a_), PDPA is soluble as a cationic polyelectrolyte, but it is insoluble in basic aqueous media. Recently, Liu et al. [27] reported the pH-dependent micellization of the ABC miktoarm star terpolymer of PEG*-b-*PMAA*-b-*PDEA. Three types of micellar aggregation were observed with the PEG/MAA hydrogen-bonded complex-core micelles at low pH values, the complexed polyelectrolyte-core micelles at intermediate pH, and the hydrophobic PDEA-core micelles at high pH.

pH-dependent micellization also applies for linear MPEG_45_*-b-*PMAA_20_*-b-*PDPA_18_ and MPEG_45_*-b-*PMAA_47_*-b-*PDPA_47_ triblock copolymers. MPEG and PMAA form a strong hydrogen-bonded complex at pH values of <4.0. In this range of pH, micelles with MPEG/PMAA complex cores were stabilized by unbounded MPEG and protonated PDPA blocks.

**Figure 3:**
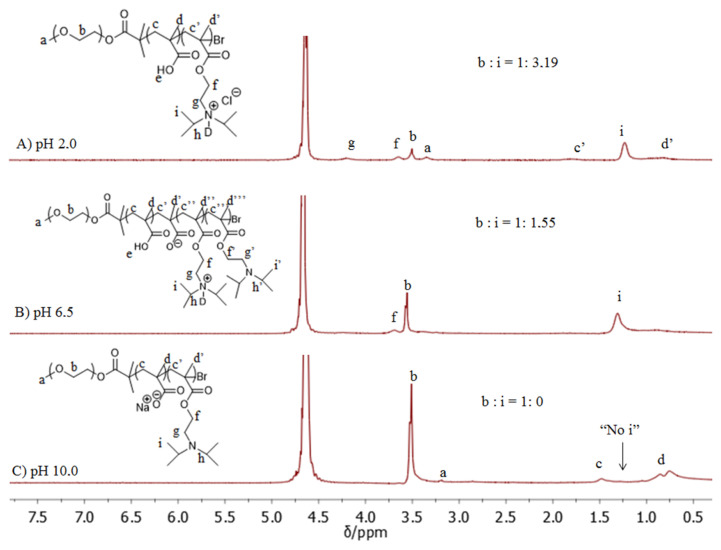
^1^H NMR spectra of MPEG_45_-*b*-PMAA_47_-*b*-PDPA_47_ triblock copolymer at different pH values in D_2_O.

**Figure 4:**
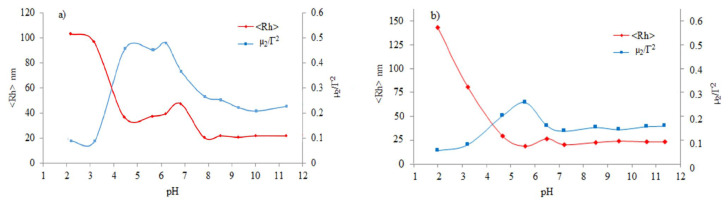
The variation of micelle hydrodynamic radii <R_h_> and polydispersity indexes at different pH values for (a) MPEG_45_*-b-*PMAA_20_*-b-*PDPA_18_ and (b) MPEG_45_*-b-*PMAA_47_*-b-*PDPA_47_ triblock copolymers.

^1^H NMR studies at pH 2 for both MPEG_45_*-b-*PMAA_20_*-b-*PDPA_18_ and MPEG_45_*-b-*PMAA_47_*-b-*PDPA_47_ systems showed that some methylene protons (–OCH_2_CH_2_O–) of the MPEG blocks were present in the NMR spectra, which is an indication of the partial interaction of the MPEG residues with PMAA residues. While the formation of micelles with the PDPA-PMAA polyion complex core is expected to occur at pH 6.5, the ^1^H NMR spectrum indicated that protons of the PDPA isopropyl groups were still visible at this pH. However, the integral ratio between the methylene protons of MPEG and the isopropyl group of PDPA is smaller than that at pH 2 (see Figure 3). This indicates that the hydrogen bonds between MPEG and PMAA being broken and the formation of the PDPA/PMAA polyion complex core started to take place. As can be seen in Figure 4, the DLS results indicated an increase in micelle diameter at pH 6.5.

**Scheme 2:**
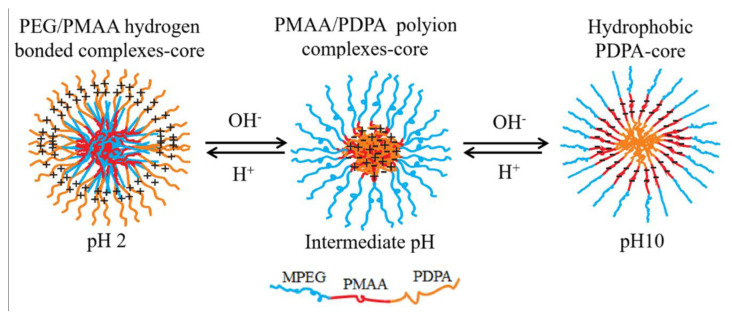
Schematic illustration for the self-assembly of MPEG-b-PMAA-b-PDPA triblock copolymers.

At pH 10, the PDPA block became fully deprotonated and gained a hydrophobic character, forming a micelle core, while the PMAA blocks were in completely ionized form and became hydrophilic. As can be seen in Figure 3, the characteristic signal of PDPA at δ 1.4 ppm completely disappeared at pH 10, but the signals of methylene protons (–OCH_2_CH_2_O–) from the backbones of the MPEG and PMAA residues were still visible. This result matched our expectation of PDPA core micelles at pH 10 stabilized by PEG/ionized PMAA hybrid blocks as hydrated coronas. A schematic illustration of PDPA-core micelle formation from the MPEG*-b-*PMAA*-b-*PDPA triblock copolymer is provided in Scheme 2.

As shown in Figure 4, the DLS studies revealed the variation of hydrodynamic radii R_h_ with solution pH at 20 °C for the micelles of MPEG_45_-*b*-PMAA_20_-*b*-PDPA_18_ and MPEG_45_-*b*-PMAA_47_-*b*-PDPA_47_ triblock copolymers, where at pH levels of >8, the <R_h_> likely remains constant at 23 nm for MPEG_45_*-b-*PMAA_47_*-b-*PDPA_47_ and 21 nm for MPEG_45_*-b-*PMAA_20_*-b-*PDPA_18_. A comparison indicated that MPEG_45_*-b-*PMAA_47_*-b-*PDPA_47_ formed considerably larger PDPA-core micelles compared to those of MPEG_45_*-b-*PMAA_20_*-b-*PDPA_18_. This was due to the higher PDPA content in MPEG_45_*-b-*PMAA_47_*-b-*PDPA_47_. Below pH 8, the <R_h_> of the triblock copolymer increased considerably for both triblock copolymers. Under these conditions, the PDPA block started to gain some positive charge due to partial protonation and the carboxylic acid residues of the PMAA block becoming ionized and gaining an anionic character. Micelles with mixed PMAA/PDPA cores and MPEG corona were formed because of these charge compensations. In Figure 4, we can see that <R_h_> increased, reaching its maximum of about 50.6 nm for MPEG_45_*-b-*PMAA_20_*-b-*PDPA_18_ in the pH range of 4.7–6.6. It was found that the aggregation behaviors of MPEG_45_*-b-*PMAA_47_*-b-*PDPA_47_ and MPEG_45_*-b-*PMAA_20_*-b-*PDPA_18_ were quite similar to each other (Figure 4a and 4b). In this case, the diameters increased dramatically at pH 8 and reached a maximum at approximately pH 6.5.

In general, zwitterionic diblock copolymers are known to be insoluble or can be precipitated at their isoelectric points [16]. In our triblock copolymer, there is a PMAA-*b*-PDPA zwitterionic diblock and neutral-hydrophilic MPEG block. The nonionic MPEG block can act as a steric stabilizer in the case of polyion complexation among anionic PMAA and cationic PDPA blocks, allowing the formation of micelles at intermediate pH levels. In the pH range of 2–4, large micellar aggregates were observed with <R_h_> = 100 nm for MPEG_45_*-b-*PMAA_20_*-b-*PDPA_18_ at pH 2 and <R_h_> = 148 for MPEG_45_*-b-*PMAA_47_*-b-*PDPA_47_. This met our expectation that micelles would be formed by MPEG/PMAA hydrogen-bonded cores via stabilization by protonated PDPA coronas. The PDI values were close to 0.1 for both micellar systems, confirming the very good reliability of the data.

### 3.3. Intermediary layer cross-linked micelles with pH-responsive core

In recent years, BIEE has been widely used as a cross-linker to cross-link PDMA residues in block copolymers to improve the stability of the micellar structure [16,25,26,29,30]. However, to cross-link PDMA residues with BIEE, a reaction of at least 3 days was needed. In our previous study, for the first time, we reported the synthesis of two types of zwitterionic shell cross-linked micelles from a PDMA-PTHPMA precursor, and cross-linking was done for type I over PDMA residues and for type II over PMAA residues (after removal of the THP group by acid hydrolysis). The reaction between the BIEE and PMAA residues gave a faster reaction time, and the reaction with the PMAA homopolymer was nearly completed within 5–10 min [26].

In the present study, we synthesized MPEG_45_*-b-*PMAA_20_*-b-*PDPA_18_ and MPEG_45_*-b-*PMAA_47_*-b-*PDPA_47_ triblock copolymers. For the synthesis of ILCL micelles, first the triblock copolymer was dissolved directly in distilled water, the appearance of which was cloudy due to aggregate formation where MPEG/PMAA complexes compromised the micelle core. By increasing the solution pH to 10, the solution color was changed from clear to bluish, indicating the formation of a kind of micelle with PDPA as a core and ionized PMAA-neutral MPEG as the corona. The shell cross-linking of the inner PMAA layer was achieved by the addition of BIEE as a bifunctional cross-linker. The cross-linking targets were 20%, 30%, and 60% based on PMAA residues. BIEE was added to the solutions by stirring at 1250 rpm. The solutions were stirred for another 2 h after BIEE was added.

**Figure 5:**
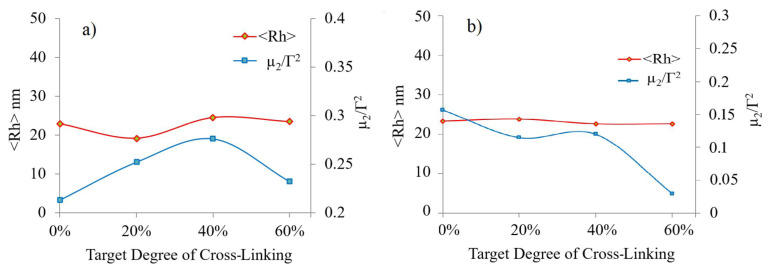
The hydrodynamic radii and polydispersity indexes of (a) MPEG_45_*-b-*PMAA_20_*-b-*PDPA_18_ and (b) MPEG_45_*-b-*PMAA_47_*-b-*PDPA_47_ ILCL micelles with various targeted degrees of cross-linking.

After cross-linking, DLS studies at pH 10 showed that the hydrodynamic radius <R_h_> for MPEG_45_*-b-*PMAA_20_*-b-*PDPA_18_ slightly increased linearly as the targeted degree increased, with <R_h_> being approximately 23 nm at targets of 40% and 60% (Figure 5a). The hydrodynamic radius <R_h_> of the MPEG_45_*-b-*PMAA_47_*-b-*PDPA_47_ micelles were relatively similar after cross-linking in the range of 22–24 nm (Figure 5b). The PDI values of the ILCL micelles at pH 10 were close to 0.25 for MPEG_45_*-b-*PMAA_20_*-b-*PDPA_18_ and close to 0.1 for MPEG_45_*-b-*PMAA_47_*-b-*PDPA_47_. The narrow PDIs suggested that the cross-linking reaction took place exclusively inside the inner PMAA layer and intermicellar cross-linking did not occur due to the steric stabilization provided by the MPEG corona.

**Figure 6:**
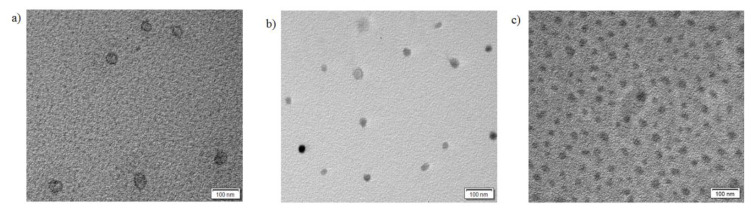
TEM images of ILCL micelles from MPEG_45_*-b-*PMAA_47_*-b-*PDPA_47_ at various targeted cross-linking degrees: (a) 20%, <D_h_> = ~35–40 nm; (b) 40%, <D_h_> = ~25–30 nm; (c) 60%, <D_h_> = ~30–35 nm.

**Figure 7:**
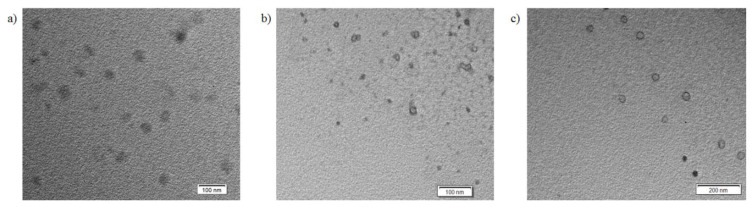
TEM images of ILCL micelles from MPEG_45_*-b-*PMAA_20_*-b-*PDPA_18_ at various targeted cross-linking degrees: (a) 20%, <D_h_> = ~ 35–40 nm; (b) 40%, <D_h_> = ~20–30 nm; (c) 60%, <D_h_> = ~40–45 nm.

TEM images of the ILCL micelles (Figure 6 and 7) revealed the presence of the micelles presumably in spherical form with diameters (D_h_) smaller than those obtained from DLS. It was to be expected that the diameters obtained from TEM were typically smaller than those obtained from DLS measurements because TEM reflected the dimensions in a dry state, while DLS studies reflected the dimensions of the stretched PEG and PMAA corona. The general agreement between DLS and TEM size results suggested that a spherical morphology like that observed by TEM was present in the solution. When we compare Figures 6 and 7, the TEM images indicate that the cross-linked micelles obtained from the polymer with higher DPA content are more monodisperse.

**Figure 8:**
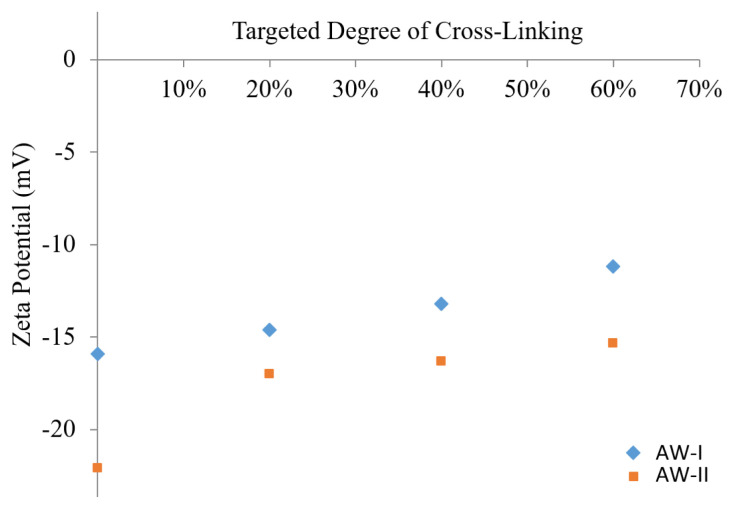
Zeta potential values of MPEG_45_*-b-*PMAA_20_*-b-*PDPA_18_ and MPEG_45_*-b-*PMAA_47_*-b-*PDPA_47_ triblock copolymer micelles and ILCL micelles at pH 9 with various targeted degrees of cross-linking.

**Figure 9:**
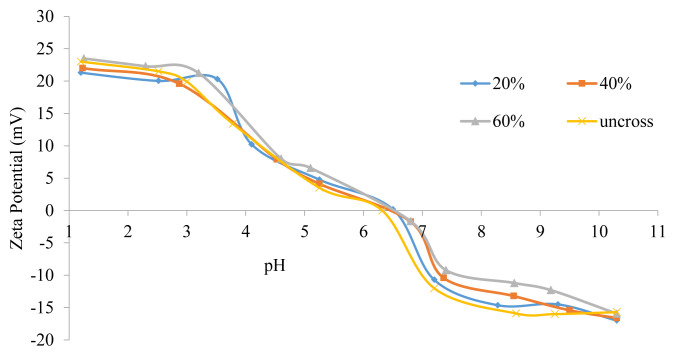
Zeta potential of MPEG_45_*-b-*PMAA_20_*-b-*PDPA_18_ ILCL micelles and uncross-linked micelles at various pH values.

Zeta potential measurements of cross-linked micelles were performed to check the occurrence of cross-linking reactions with various targeted degrees by comparison with precursor micelle zeta potentials at pH 9 (see Figure 8). The results satisfied our expectations as the zeta potential increased linearly with increasing degrees of cross-linking. The increased zeta potential can be explained by the loss of the negative charge from the PMAA residue because of the esterification reaction between the PMAA and BIEE. The higher the target, the more PMAA will react with BIEE, causing more negative charge to be lost (Figures 8 and 9).

### 3.4. Drug loading and in vitro drug release

Hydrophobic drugs can be loaded into the micelles due to presence of the hydrophobic core in the micelles. DIP is a coronary vasodilator drug with very low solubility in basic aqueous media. It is soluble below pH 5.9 due to the protonation of amine groups. It is insoluble above pH 5.9 and precipitates as yellow, needle-shaped crystals [31]. Thus, DIP and the triblock copolymer were first dissolved molecularly in acidic media and then the solution pH was increased above the pH where micellization occurred. Under micellar conditions, water-insoluble DIP could be localized in the hydrophobic core of the micelles. By using DIP as a model drug, the controlled release properties of MPEG_45_*-b-*PMAA_20_*-b-*PDPA_18_ and MPEG_45_*-b-*PMAA_47_*-b-*PDPA_47_ triblock copolymer micelles were evaluated. The obtained CLMs offer potential as drug carriers due to the hydrophilic and biocompatible PEG corona and the pH-responsive PDPA core.

**Scheme 3:**
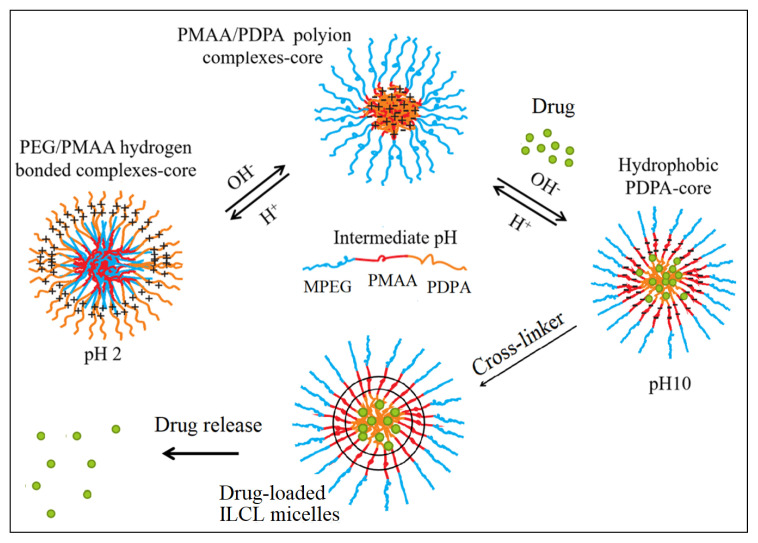
Formation of micelles, drug-loaded cross-linked micelles, and drug release.

The DIP and copolymer were mixed in water at pH 3 and DIP was loaded to the micelle cores by adjusting the solution pH to 10 (see Scheme 3). The DIP loading capacity of the micelles was calculated to be 5.9 mg/g and 9.7 mg/g for MPEG_45_*-b-*PMAA_20_*-b-*PDPA_18_ and MPEG_45_*-b-*PMAA_47_*-b-*PDPA_47_, respectively. It is notable that the longer PDPA chain can provide more drug contents. The DLS studies of drug-loaded micelles showed that the size of the micelles increased after drug encapsulation. As determined via DLS studies, the average hydrodynamics radius <R_h_> of the micelles was 22.7 and 28.9 nm for MPEG_45_*-b-*PMAA_20_*-b-*PDPA_18_ and MPEG_45_*-b-*PMAA_47_*-b-*PDPA_47_, respectively.

After loading the drugs into the micelles, the solutions were divided into four parts of 10 mL each, and then cross-linking of the micelle intermediary layer was done at 0%, 20%, 40%, and 60% based on PMAA residues. The drug release profiles based on different targeted degrees of cross-linking were monitored using the dialysis method. The amounts of DIP released into the external buffer solution were determined at a fixed pH of 3.0 and wavelength of 283.52 nm based on the standard curve of DIP.

**Figure 10:**
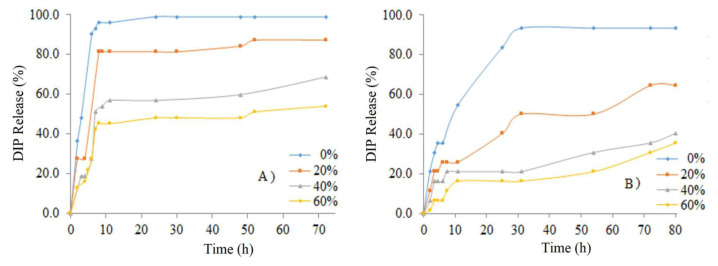
Cumulative DIP release at 37 °C from micelles and cross-linked micelles of (a) MPEG_45_*-b-*PMAA_20_*-b-*PDPA_18_ and (b) MPEG_45_*-b-*PMAA_47_*-b-*PDPA_47_ triblock copolymer.

Figure 10 shows the time dependence of the cumulative DIP release of drug-loaded ILCL micelles into buffer solutions with different cross-linking degrees. When the drug-loaded uncross-linked micelle and ILCL micelle solutions were placed in a buffer of pH 7.4 at 37 °C, only about 50% of the loaded drug could be released from the ILCL micelles with a targeted degree of 60% even after 80 h (see Figure 10a). A buffer solution of pH 7.4 was used, in which the PDPA core became more hydrophobic. The hydrophobic character of the PDPA blocks gave better solubilization for hydrophobic drugs; thus, they would likely remain in the micelle cores. However, cross-linking may also partially retard the drug release. As we can see in Figure 10, the increase of the cross-linking degree retarded the drug release from the micelle cores. In a buffer solution of pH 7.4 and at 37 °C, about 99% of the DIP was released from uncross-linked micelles within 10 h, but only 84%, 46%, and 37% of the drug was released from the ILCL micelles of MPEG_45_*-b-*PMAA_20_*-b-*PDPA_18_ with targeted degrees of cross-linking of 20%, 40%, and 60%, respectively (Figure 10a). On the other hand, a similar amount of DIP was released from the uncross-linked micelles of the MPEG_45_*-b-*PMAA_47_*-b-*PDPA_47_ triblock copolymer within 30 h (Figure 10b). Additionally, less and more controlled drug release was observed with the ILCL micelles of the MPEG_45_*-b-*PMAA_47_*-b-*PDPA_47_ triblock copolymers compared to the release profile of MPEG_45_*-b-*PMAA_20_*-b-*PDPA_18_-based ILCL micelles.

### 3.5. Conclusions

pH-responsive ABC-type zwitterionic triblock copolymers (MPEG*-b-*PMAA*-b-*PDPA) were synthesized in this study using ATRP followed by acid hydrolysis. By changing the solution pH, the MPEG-*b*-PMAA-*b*-PDPA triblock copolymer can form three types of micellar aggregates in which the main driving forces are hydrogen bonding, polyion complexation, and hydrophobic interactions. Intermediary layer cross-linked PDPA-core micelles can be obtained in 2 h using BIEE as a cross-linker, which reacts well with the PMAA block at pH 10 and 20 °C. Hydrophobic drugs such as DIP can be encapsulated in the micelle cores at high pH values. When the cross-linking degree is increased, the drug release gets slower. Therefore, the drug release rate can be adjusted by varying the degree of cross-linking. This ABC-type triblock copolymer has good potential as a drug carrier and release system for biomedical applications.

## Figures and Tables

**Table:** Summary of the synthesized triblock copolymers

Code	Composition	Precursor *M_n_* (g/mol)*	DP**	*M_n_* (g/mol) **	Precursor M_w_/M_n_*
AW-I	MPEG*-b-*PMAA*-b-*PDPA	9650	45/20/18	7710	1.15
AW-II	MPEG*-b-*PMAA*-b-*PDPA	12940	45/47/47	16220	1.22

* As determined by GPC analysis.

** As calculated from proton NMR spectra of precursor triblock copolymers.
